# Rethinking Vitamin A Deficiency: Its Causes, Ophthalmologic Presentation, and Management Gaps at a New England Tertiary Hospital

**DOI:** 10.3390/nu18081310

**Published:** 2026-04-21

**Authors:** Katherine H. Fearon, Corbin M. Dameron, Shannon L. Kelleher, Amer Al-Nimr, Michael E. Zegans

**Affiliations:** 1 Department of Surgery, Section of Ophthalmology, Dartmouth-Hitchcock Medical Center, Lebanon, NH 03756, USA; katherine.h.fearon@hitchcock.org (K.H.F.); corbin.m.dameron.med@dartmouth.edu (C.M.D.); 2 Department of Biomedical and Nutritional Sciences, University of Massachusetts Lowell, Lowell, MA 01854, USA; shannon_kelleher@uml.edu; 3 Department of Pediatric Gastroenterology, Dartmouth-Hitchcock Medical Center, Lebanon, NH 03756, USA; amer.al-nimr@hitchcock.org

**Keywords:** vitamin A, xerophthalmia, zinc, retinol binding protein, malnutrition

## Abstract

**Background/Objectives**: To evaluate ocular disease and eye care utilization among adults with vitamin A deficiency (VAD) in a high-resource healthcare setting, with particular emphasis on nutritional etiologies, clinical nutrition oversight, and outcomes associated with severity of deficiency. **Methods**: A retrospective chart review was conducted at Dartmouth Hitchcock Medical Center (DHMC) from 1 January 2019 through 31 December 2022. Adults (>18 years) with measured serum retinol concentrations were identified, and data were extracted on retinol concentration, diagnosis, referring service, and vital status. Patients with VAD (serum retinol <32.5 µg/dL per our laboratory threshold) underwent detailed chart review, including social determinants of health and documented nutritional risk factors. For patients with VAD who received an ophthalmologic evaluation, slit lamp findings, ocular symptoms, duration of deficiency, and vitamin A treatment were assessed. **Results**: VAD was identified in 752 of 2725 patients (27.7%) tested for VAD, and 330 patients had concentrations below the World Health Organization (WHO) threshold for VAD (<20 µg/dL). Hepatic, nutritional, and malabsorptive conditions were prominent contributors, including cirrhosis related to alcohol use or hepatitis C virus (30%), malnutrition or malabsorption following bariatric surgery (24%), and pancreatic insufficiency (20.1%). Food insecurity data were incomplete but showed no significant association with vitamin A concentration. Despite biochemical evidence of deficiency, only 72 patients with VAD (9.6%) underwent ophthalmologic evaluation, and only three were referred specifically due to VAD. Clinical signs or symptoms consistent with xerophthalmia were observed in 21% of those evaluated, and 18% demonstrated corneal findings. Vitamin A supplementation was documented in just over half of symptomatic patients, with objective or symptomatic improvement noted in three cases. VAD was explicitly acknowledged in only 9.7% of ophthalmology notes. Increasing severity of VAD was strongly associated with mortality (*p* < 0.001), independent of food insecurity, which showed no association with serum retinol concentrations. **Conclusions**: In this high-resource clinical setting, VAD is common in an at-risk population and largely driven by nutrition-related disease states affecting absorption, metabolism, and hepatic storage. Despite clear biochemical deficiency and associated mortality risk, VAD is underrecognized, undertreated, and infrequently linked to ocular evaluation, highlighting a critical gap in nutrition-focused screening, interdisciplinary communication, and proactive vitamin A assessment in medically complex adults.

## 1. Introduction

Vitamin A (VA) and its retinoid metabolites are essential nutrients with critical roles in vision, epithelial integrity, and gene regulation [1,2,3,4]. Retinaldehyde serves as the chromophore required for phototransduction, while retinoic acid regulates transcriptional programs essential for ocular surface maintenance and immune function [1,2,3,4]. Consequently, vitamin A deficiency (VAD) disrupts multiple visual and epithelial processes essential for ocular surface maintenance and immune function [1,2,3,4]. As a result, VAD can lead to xerophthalmia, a spectrum of ocular manifestations including conjunctival and corneal xerosis, Bitot’s spots, keratomalacia, nyctalopia, and retinopathy [2,5].

In the United States, preformed retinoids and provitamin A carotenoids are widely available through animal and plant-based foods, and overt dietary insufficiency is considered uncommon. As a result, VAD is most often associated with children and pregnant or lactating individuals living in low-resource settings where malnutrition predominates [1,2,6]. However, emerging case reports and small series demonstrate that VAD also occurs in high-resource clinical settings, particularly in the context of chronic disease, malabsorption, altered hepatic storage, or impaired nutrient metabolism [7,8,9,10,11]. These secondary forms of VAD are likely underrecognized in clinical practice.

Following a case series of three adults presenting with xerophthalmia at Dartmouth Hitchcock Medical Center (DHMC), a review of all serum retinol measurements obtained at DHMC in that year identified 431 patients with biochemical VAD, including 158 with serum retinol concentrations comparable to those observed in xerophthalmic cases [7]. The unexpectedly large number of nutritionally vulnerable adults identified in this setting prompted a broader evaluation of VAD prevalence, associated comorbidities, and ocular outcomes in an at-risk population.

The purpose of this study was to characterize the demographic distribution, nutrition-related comorbidities, and ocular disease burden among adults with VAD at DHMC between 2019 and 2022. By situating VAD within the context of secondary micronutrient deficiency in medically complex populations, this work aims to highlight gaps in nutrition screening, risk recognition, and interdisciplinary management in high-resource healthcare systems.

## 2. Materials and Methods

This retrospective study was conducted in accordance with the Declaration of Helsinki and the Health Insurance Portability and Accountability Act and was approved by the Dartmouth–Hitchcock Health Human Research Protection Program (IRB #02002110). Adult patients (≥18 years) with at least one serum retinol measurement obtained at Dartmouth Hitchcock Medical Center (DHMC) between 1 January 2019 and 31 December 2022 were eligible for inclusion. Individuals who were pregnant, incarcerated, or <18 years of age were excluded.

Nutrition and health status—A detailed chart review was performed for patients with VAD to characterize nutrition-related comorbidities and clinical outcomes. Serum retinol concentrations were classified using the clinical laboratory reference threshold of <32.5 µg/dL (1.13 µmol/L) to define low vitamin A status, as reported in the electronic medical record. This threshold is higher than the World Health Organization (WHO) threshold for VAD (20 µg/dL = 0.70 µmol/L). For patients with VAD, demographic data, serum retinol, zinc, and total protein concentrations, and diagnoses associated with vitamin A testing were extracted from the electronic medical record. When multiple serum retinol measurements were available, the lowest value was used for analysis to reflect maximal nutritional deficiency and capture clinically significant VAD in this population. For patients evaluated by ophthalmology, exam notes and slit-lamp findings were reviewed. Data abstraction was performed independently by two reviewers to ensure accuracy and consistency.

Nutrition concentrations—Serum retinol concentrations were measured using liquid chromatography–tandem mass spectrometry (LC-MS/MS). Serum zinc concentrations were measured using triple-quadrupole inductively coupled plasma mass spectrometry (ICP-MS/MS). Serum protein concentrations were measured using a colorimetric biuret assay, conducted using an automated clinical chemistry analyzer. All laboratory values were obtained from the electronic medical record.

Food insecurity—Food insecurity was assessed using the Hunger Vital Sign Screening Tool (Hunger Vital Sign™ 2017), a validated two-item instrument with high sensitivity (97%) and specificity (83%), and incorporated into the Centers for Medicare & Medicaid Services Accountable Health Communities Screening Tool [12,13].

Statistics—Statistical analyses were performed using Stata/SE version 18.0 (StataCorp, College Station, TX, USA). Serum retinol distributions were assessed for normality using the Shapiro–Wilk test. Nonparametric comparisons across multiple groups were conducted using the Kruskal–Wallis test with post hoc Dunn’s tests and Bonferroni correction. Pairwise comparisons were performed using the Mann–Whitney U test. Associations between categorical variables were assessed using Pearson’s chi-square test, and correlations were evaluated using Spearman coefficients. Statistical significance was defined as *p* < 0.05.

## 3. Results

**Figure 1:**
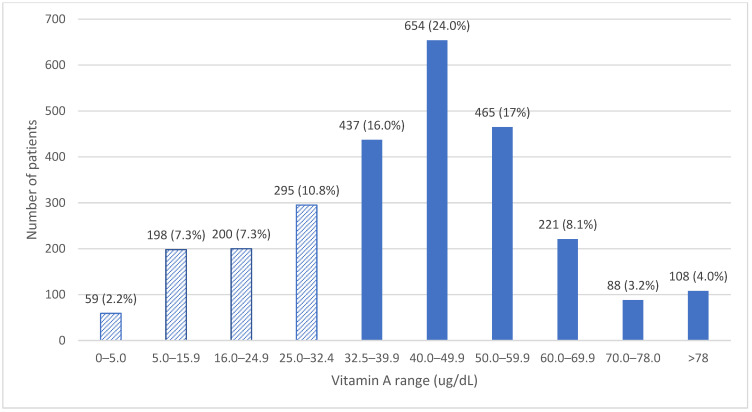
Distribution of serum vitamin A concentrations among adults tested at Dartmouth Hitchcock Medical Center from 1 January 2019 to 31 December 2022 (n = 2725). Serum retinol concentrations were undetectable in 59 patients; 198 had concentrations of 5.0–15.9 µg/dL, 200 had 16.0–24.9 µg/dL, and 295 had 25.0–32.4 µg/dL. Normal vitamin A concentrations (32.5–78.0 µg/dL) were observed in 1865 patients (dark blue), and 108 patients had concentrations >78.0 µg/dL (pale blue).

**Figure 2:**
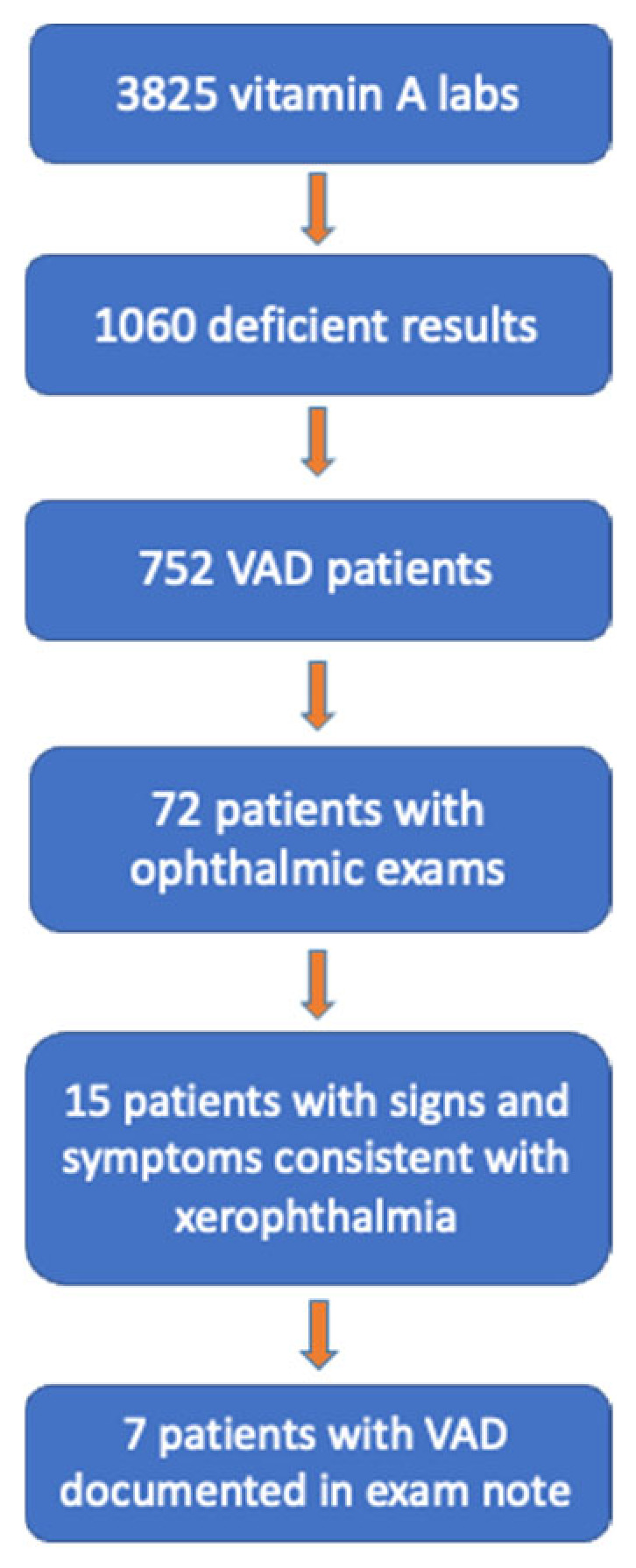
Flow diagram illustrating cohort selection and identification of patients with VAD–related ocular findings. Of 3825 vitamin A laboratory tests, 1060 results from 752 patients returned deficient. Among these, 72 had documented ophthalmic examinations, 15 exhibited signs and symptoms consistent with xerophthalmia, and 7 had VAD documented on ophthalmic examination.

Between 2019 and 2022, 3825 serum vitamin A (VA) tests were ordered for 2725 adult patients. Vitamin A deficiency (VAD; serum retinol <32.5 µg/dL) was identified in 752 patients (27.7%) (Figure 1). Only 72 patients with VAD (9.6%) underwent ophthalmologic evaluation. Among those evaluated, 21% exhibited signs or symptoms consistent with xerophthalmia, most commonly corneal findings (Figure 2).

Serum retinol concentrations were most frequently ordered by gastroenterology, general surgery, family medicine, pulmonary disease, surgical oncology, and hematology, reflecting testing predominantly in medically complex populations.

### 3.1. Etiology

The most common conditions associated with VAD were liver cirrhosis (30%), malnutrition or malabsorption following bariatric surgery (24%), pancreatic insufficiency (20.1%), and documented malnutrition (10%) (Table 1). Serum retinol concentrations differed significantly by disease category, with the lowest concentrations observed in patients with alcohol- or hepatitis C virus–associated cirrhosis (Kruskal–Wallis *p* < 0.001, Dunn’s pairwise comparison *p* < 0.01).

Food insecurity screening was completed in a subset of patients (n = 100). While 27% screened positive for food insecurity, serum retinol concentrations did not differ between food-secure and food-insecure groups.

### 3.2. Repeat Testing

**Figure 3:**
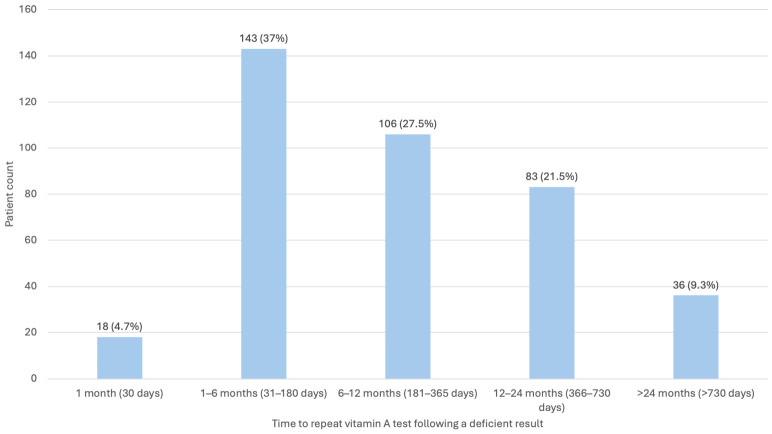
Time to repeat Vitamin A test (n = 386 patients). Of 752 VAD patients, 386 underwent repeat VA testing after a deficient result. 18 had a repeat test within 1 month of their first deficient result. 143 had repeat testing within 1–6 months, 106 within 6–12 months, 83 within 12–24 months, and 36 over 24 months.

Forty-three percent of patients with VAD had only one serum retinol measurement. Among those with repeat testing (51%), the median interval between initial and follow-up testing was 211 days, with most repeat tests occurring within 12 months (Figure 3). On repeat testing, 61% of patients remained deficient. Among those who normalized serum retinol concentrations, resolution occurred after a median of 258 days.

### 3.3. Ocular Findings

**Figure 4:**
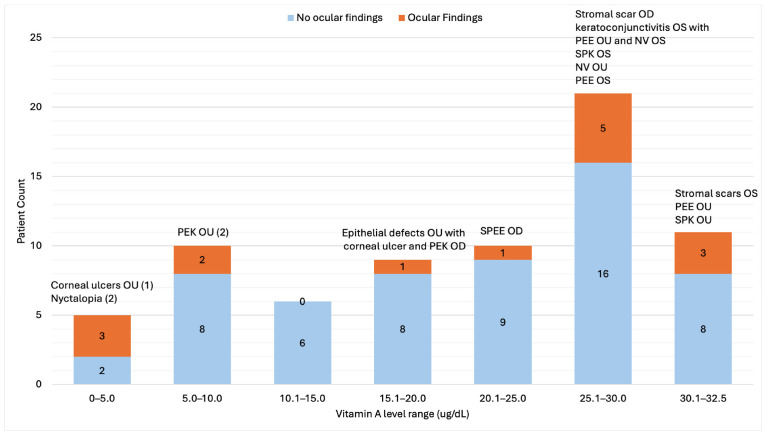
Spectrum of vitamin A values and ocular findings of VAD patients with ophthalmic exams. Ocular findings included corneal ulcers, nyctalopia, epithelial defects, punctate epithelial keratitis (PEK), superficial punctate epithelial erosions (SPEE), punctate epithelial erosions (PEE), neovascularization (NV), keratoconjunctivitis, superficial punctate keratitis (SPK), and scarring.

**Figure 5:**
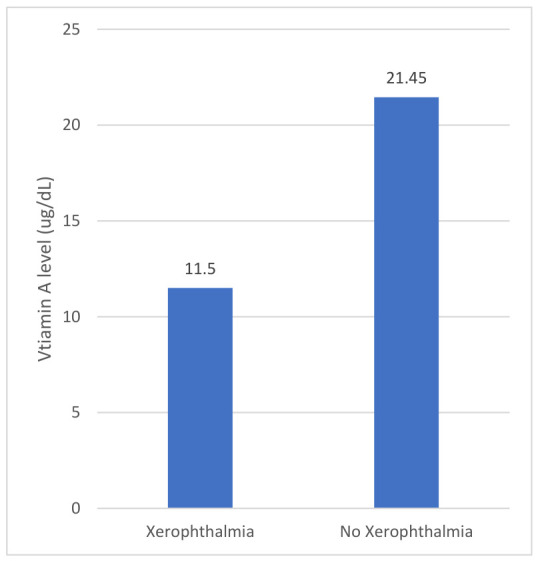
Mean Vitamin A concentration of patients with versus without xerophthalmia (n_xerophthalmia_ = 8; n_no xerophthalmia_ = 64). The average VA of patients with xerophthalmia was 11.5 µg/dL in contrast to 21.5 µg/dL among patients without (z = 2.51, *p* = 0.01).

Among patients with VAD, 9.6% were evaluated by ophthalmology, with only three referred specifically for suspected VAD. Corneal abnormalities (ulcers, epithelial defects, stromal scars, punctate epithelial erosions or keratitis) were identified in 18% of evaluated patients (Figure 4), and xerophthalmia-consistent findings were associated with significantly lower serum retinol concentrations compared with those without ocular involvement (11.5 vs. 21.5 µg/dL, two-sample Wilcox rank-sum test, z = 2.51, exact *p* = 0.01) (Figure 5). VAD was explicitly documented in fewer than 10% of ophthalmology notes. Among symptomatic patients who received VA supplementation, improvement in ocular findings was documented in select cases.

Night blindness was reported by two patients, and dry eye was documented in 18 patients. Vitamin A deficiency was explicitly noted in ophthalmology documentation for only 7 of 72 patients (9.7%). Pre-supplementation serum retinol concentrations in these patients ranged from undetectable to 29.9 µg/dL. Among patients with ocular findings consistent with xerophthalmia, 8 of 15 received vitamin A supplementation, with documented improvement in ocular findings in 3 of 5 patients in whom VAD was acknowledged in the clinical record.

### 3.4. Other Nutritional Results

**Figure 6:**
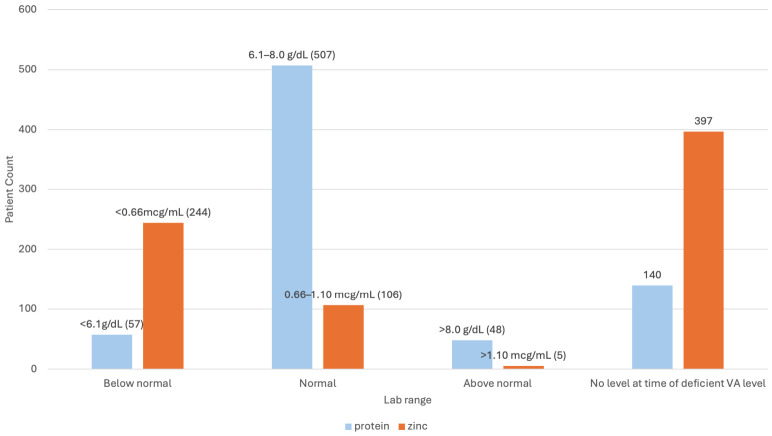
Protein and Zinc Concentrations of VAD Patients (n_protein_ = 612 patients; n_zinc_ = 355 patients). Of 612 VAD patients with protein concentrations assessed at the time of VAD, 57 were protein-deficient (<6.1 g/dL), 507 had normal protein (6.1–8.0 g/dL), and 48 had hyperproteinemia (>8.0 g/dL). Of 355 patients with zinc concentrations assessed at the time of VAD, 244 were zinc deficient (<0.66 mcg/mL), 106 had normal zinc (0.66–1.10 mcg/mL), and 5 had elevated zinc concentrations (>1.10 mcg/mL). 140 and 397 patients lacked protein or zinc data, respectively.

Most patients with concurrent protein testing had normal total protein concentrations; however, protein deficiency was associated with significantly lower serum retinol concentrations (Spearman rank correlation, *p* < 0.0001). Zinc deficiency was common (69%) among those tested and was also associated with lower serum retinol concentrations (Spearman rank correlation, *p* < 0.0001) (Figure 6).

### 3.5. Mortality

Serum retinol concentration was strongly associated with mortality. Long-term mortality increased stepwise with VAD severity, from 8.5% in mild deficiency (25–32.5 µg/dL); 26% in moderate deficiency (16–25 µg/dL); 36.4% in profound VAD (5–16 µg/dL); to 42.4% in patients with undetectable retinol (<5.0 µg/dL) (Pearson Chi squared test, *p* < 0.001). Patients who died had significantly lower serum retinol concentrations than survivors (14.4 ± 8.4 vs. 21.3 ± 8.9 µg/dL; Mann–Whitney U Test, U = 47873.0, z = 8.75, *p* < 0.001).

## 4. Discussion

Vitamin A deficiency (VAD) remains a major cause of morbidity and mortality in low-resource settings [14,15,16,17], yet our findings demonstrate that VAD is also prevalent in a high-resource U.S. healthcare system. Over a four-year period, we identified 752 adults with biochemical VAD at a tertiary medical center in New England, including patients with ocular findings consistent with xerophthalmia. There are limited data documenting VAD rates in adult populations in well-resourced settings. The prevalence observed in our cohort of patients tested for VAD (27.7%) is higher than previously reported rates, including one other study from a well-resourced setting that reported a VAD prevalence of 20%, although this estimate was based on all laboratory tests performed rather than unique patients [9]. These data underscore that VAD in high-income hospital settings is not rare and is primarily driven by disease-associated mechanisms rather than inadequate food access.

Consistent with prior reports from high-resource settings [7,8,9,10,11], VAD in our cohort was strongly associated with liver disease, bariatric surgery, and pancreatic insufficiency. Patients with alcohol- or hepatitis C virus–related cirrhosis exhibited the lowest serum retinol concentrations, likely reflecting combined effects of poor intake, impaired hepatic storage, and defective mobilization of vitamin A [18,19]. In contrast, patients with prior bariatric surgery had higher mean retinol concentrations overall, likely due to guideline-driven nutritional monitoring [20], yet demonstrated a wide range of deficiency severity. The frequency and persistence of VAD in this group, even years after surgery, highlight bariatric surgery as a predictable and ongoing risk requiring long-term micronutrient surveillance [21,22].

While the acute phase response can also be associated with a transient drop in vitamin A concentration following trauma, infection, or stress, the median time to VAD resolution among patients with repeat testing was 211 days, suggesting the majority of cases were chronic rather than acute disease processes [23]. Future studies including analysis of CRP and inflammatory marker data will be important in better classifying the role of the acute phase response in VA status in a well-resourced environment.

Despite the disease-associated origin of VAD in this population, clinical recognition and follow-up were inconsistent. Most patients with biochemical VAD did not undergo ophthalmologic evaluation, even when serum retinol concentrations were comparable to those associated with xerophthalmia. Among those evaluated, documentation of VAD was uncommon, suggesting limited integration of nutritional data into specialty care. Importantly, lower serum retinol concentrations were associated with ocular findings consistent with xerophthalmia, reinforcing the biological relevance of biochemical deficiency even in medically complex adults.

Our data further highlight the importance of micronutrient interactions in VAD. Zinc deficiency was highly prevalent and strongly associated with lower serum retinol concentrations. Zinc is required for retinol binding protein synthesis and retinol metabolism [24,25], and deficiency may contribute to functional VAD even when serum retinol appears near normal. Given that zinc deficiency commonly co-occurs with cirrhosis, bariatric surgery, and pancreatic insufficiency [18,26,27], concurrent assessment of zinc status may be essential for effective VAD management. Evidence from pediatric populations suggests that combined zinc and vitamin A supplementation is more effective than vitamin A alone [28], though this strategy remains understudied in adults with secondary VAD.

We also observed a strong association between VAD severity and mortality. Given the severity of illness among patients with VAD in our population, mortality is likely impacted by confounding variables—such as cirrhosis, malignancy, and overall disease burden—and driven by underlying disease states rather than VAD itself. While vitamin A supplementation (VAS) reduces morbidity and mortality in undernourished pediatric populations [29,30,31], its role in disease-associated VAD remains poorly defined. In adults with chronic liver disease, VAS may be contraindicated due to hepatotoxicity risk [19,32,33], whereas in bariatric surgery and pancreatic insufficiency, supplementation is generally safe but requires careful monitoring to avoid toxicity [34,35,36,37,38]. These findings support the need for individualized, nutrition-led management strategies rather than uniform supplementation approaches.

This study has limitations, including its retrospective design, incomplete food insecurity screening, and heterogeneous supplementation practices, which precluded causal inference regarding mortality or treatment efficacy. Ocular findings consistent with xerophthalmia were observed, but there was limited follow-up assessing the response of these findings to VAS in most cases. Many of these ocular findings were nonspecific and may have been influenced by other underlying ocular diseases, which precludes causal conclusions. Additionally, retinol binding protein and iron status, both relevant to vitamin A metabolism, were infrequently assessed. CRP and other markers of inflammation were not evaluated; therefore, we cannot comment on the role of inflammation in vitamin A status. Further research including inflammatory markers will be important for classifying the role of the acute phase response in vitamin A status in well-resourced environments. Finally, our threshold used to define low serum retinol in this study was based on institutional laboratory reference values, which are higher than the cutoff established by the World Health Organization for VAD. This higher threshold likely reflects “at-risk” suboptimal VA status compared to a disease cutoff. As a result, our definition likely captures a broader group of individuals with low vitamin A status rather than strictly those meeting WHO criteria for deficiency. This difference should be considered when comparing our findings to studies using WHO-based definitions. Nonetheless, this work provides one of the most comprehensive descriptions of VAD prevalence, etiology, and outcomes in a high-resource U.S. medical center.

In summary, VAD is common, underrecognized, and clinically consequential in adults with chronic disease in high-resource settings. Liver disease, bariatric surgery, and pancreatic insufficiency represent predictable risk states warranting proactive, longitudinal micronutrient monitoring. Integration of vitamin A and zinc assessment into routine nutrition care pathways may improve recognition, guide safer supplementation strategies, and reduce preventable morbidity associated with secondary VAD.

## Figures and Tables

**Table 1:** Patient count, mean age, and mean vitamin A concentration based on diagnosis.

Etiology	Number of Patients (n = 752)	Mean Vitamin A (µg/dL)	Mean Age (years)	Sex	
				Female n (%)	Male n (%)
All Liver Disease	303	13.4 ± 8.4	60 ± 12.2	127 (41.9)	176 (58.1)
Cirrhosis due to alcohol or HCV	226	12.1 ± 7.9	58 ± 11.4	81 (35.8)	145 (64.2)
MASLD	50	17.2 ± 8.2	68 ± 9.8	30 (60)	20 (40)
Other Liver Disease	27	17.0 ± 9.6	66 ± 13.5	16 (59)	11 (41)
Bariatric Surgery	181	26.1 ± 5.0	47 ± 11.9	167 (92.3)	14 (7.7)
Pancreatic Insufficiency	151	21.8 ± 8.0	52 ± 19.6	74 (49)	77 (51)
Cystic Fibrosis	58	21.8 ± 8.1	32 ± 8.3	32 (55)	26 (45)
Malnutrition Unspecified	74	22.7 ± 8.1	56 ± 18.7	47 (64)	27 (36)
Unknown/Other	43	24.0 ± 8.0	51 ± 18.5	27 (63)	16 (37)
*p*-value		<0.001	<0.001	<0.001	

Values are presented as mean or n (%). *p*-values represent comparisons across all etiologic groups listed in the table. *p*-values calculated using Kruskal–Wallis tests for continuous variables and Pearson’s chi-square test for categorical variables.

## Data Availability

The data described in the manuscript will be made available upon request due to privacy restrictions, pending application and approval.
